# Supplementary material for: Th2 Cytokines Affect the Innate Immune Barrier without Impairing the Physical Barrier in a 3D Model of Normal Human Skin
Source: J Clin Med. 2023 Mar 1;12(5):1941. doi: 10.3390/jcm12051941 (PMC10003590; doi:10.3390/jcm12051941)
Supplement: Supplementary file 1 [file jcm-12-01941-s001.zip › jcm-2234834-supplementary.pdf]

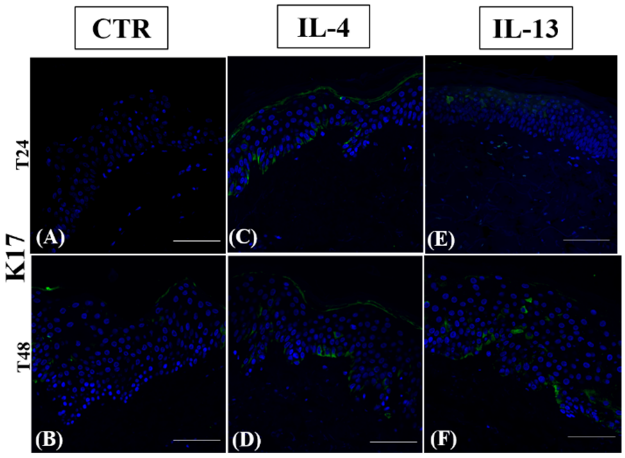

Figure S1. K17 immunofluorescence analysis. Representative K17 immunostainings in normal human skin paraffin sections. (A, C, E): samples harvested at 24 h; (B,D, F): samples harvested at 48 h. (A, B): CTR samples; (C, D): IL-4-treated samples; (E, F): IL-13-treated samples. Nuclei are counterstained with DAPI. K17: keratin 17. CTR: control; IL-4: interleukin 4; IL-13: interleukin 13; DAPI: 4′, 6-diamidino-2-phenylindoledihydrochloride. Scale bars: 100 µm.
